# Supplementary material for: Continuity and sustainability of care in family medicine: Assessing its association with quality of life and health outcomes in older populations—A systematic review
Source: PLoS One. 2024 Dec 23;19(12):e0299283. doi: 10.1371/journal.pone.0299283 (PMC11666006; doi:10.1371/journal.pone.0299283)
Supplement: S1 Dataset — https://doi.org/10.6084/m9.figshare.25465216. (DOCX) [file pone.0299283.s001.docx]

S1 Dataset. the extraction table of the included strategy

| **Study** | **Study Design** | **Diagnosis** | **Intervention** | **Control** | **Outcomes** | **Key Findings** |
| --- | --- | --- | --- | --- | --- | --- |
| Grace Sum, et al. (2021)(65) | Prospective single-arm pre-post study | Older adults with complex needs | Patient-Centered Medical Home (PCMH) model implementation | None | 1. Health-related Quality of Life (QoL) assessed by EQ-5D-5L and Visual Analog Scale (EQ VAS) 2. Needs satisfaction (CASP-19 total score) 3. Patient activation (PAM-13) | Preliminary results show no significant change in EQ-5D-5L Index and EQ VAS score at 6 months. Marginal improvements in CASP-19 total score at 3 and 6 months post-enrolment, with a significant impact on the Pleasure domain at 6 months. Improved patient activation, with a decrease in lower-level PAM participants and an increase in higher-level PAM participants from 3 to 6 months post-enrolment. |
| Zulman, et al. (2019)(58) | Cross-sectional analysis of patient survey data from a five-site randomized quality improvement study. | Patients with medical, behavioral, and social complexity at high risk of hospitalization (hospitalization risk scores ≥ 90th percentile and recent acute care). | PACT-Intensive Management (PIM) program offering intensive care coordination, including home visits, accompaniment to specialists, acute care follow-up, and case management from a team staffed by primary care providers, social workers, psychologists, nurses, and/or other support staff. | Standard VA medical home (Patient Aligned Care Team, PACT). | Patient-reported experiences with care coordination (e.g., health goal assessment, test and appointment follow-up, Patient Assessment of Chronic Illness Care (PACIC)), access to healthcare services, provider relationships, and satisfaction. | PIM patients were more likely than PACT patients to report being asked about their health goals (AOR = 1.26; P = 0.046) and having a VA provider they trust (AOR = 1.35; P = 0.005). PIM patients had higher mean PACIC scores compared with PACT patients (2.91 vs. 2.75; P = 0.022) and were more likely to report full satisfaction with primary care (AOR = 1.25; P = 0.048). Other effects on coordination, access, and satisfaction were not statistically significant. |
| Nyweide, et al. (2014)(57) | Retrospective cohort study | Preventable hospitalizations | Higher continuity of ambulatory care (measured using the continuity of care score and usual provider continuity score) | N/A | The rate of preventable hospitalization and mortality rates | Of the 3,276,635 eligible patients, 12.6% had a preventable hospitalization during the 2-year observation period. A 0.1 increase in continuity of care score was associated with about a 2% lower rate of preventable hospitalization for both continuity metrics. Continuity of care was not related to mortality rates. |
| Asma, et al.(2013)(66) | Experimental | Heart Failure | 1. One hour face-to-face education at discharge and an educational booklet.<br>2. Telephone follow-up by nurse for the next 3 months (second intervention group). | Hospital routine care | Readmission levels after 3 months (hospital, clinical, and doctor's office readmissions). | Both patient education during discharge and telephone follow-up significantly decreased readmission levels for all types of readmissions with significant differences noted in hospital, clinical, and doctor's office readmissions (p=0.06, p<0.001, p<0.001 respectively). Follow-up after discharge is effective in reducing readmissions. |
| Li Yang et al., 2022(56) | Randomized controlled trial | Alzheimer's Disease | Comprehensive intervention for 6 months, including various sectoral approaches in a nursing home | Routine interventions in a nursing home | Communication ability, housework handling ability, self-care ability, and life quality of patients; quality of life and caring ability of caregivers | After 6 months, the intervention group showed significant improvement in the life quality of patients and their caregivers compared to the control group. The communication ability, housework handling ability, self-care ability of patients, and the quality of life and caring ability of caregivers were significantly improved. |
| Bayliss et al., 2015(55) | Retrospective Cohort | Seniors with 3 or more chronic medical conditions | Continuity of Care (COC) Index | None | Inpatient admissions and emergency department visits | Greater continuity of both primary and specialty care was associated with lower risk of inpatient admissions (primary care COC hazard ratio = 0.97, specialty care COC hazard ratio = 0.95) and emergency department visits (primary care COC hazard ratio = 0.97, specialty care COC hazard ratio = 0.98). For patients with 3 or more primary and specialty care visits, specialty care continuity independently decreased the risk of inpatient admissions (hazard ratio = 0.94), and primary care continuity independently decreased the risk of emergency department visits (hazard ratio = 0.98). The study concluded that in an integrated delivery system with high informational continuity, greater continuity of care is independently associated with lower hospital utilization for seniors with multiple chronic medical conditions. |
| Halima Amjad et al., 2016(54) | Observational Retrospective Cohort | Dementia (fee-for-service Medicare beneficiaries) | Continuity of care score measured on patient visits across physicians over 12 months. | None (Observational study) | All-cause hospitalization, ambulatory care sensitive condition hospitalization, emergency department visit, imaging and laboratory testing (CT scan of the head, chest radiography, urinalysis, and urine culture), and health care spending (overall, hospital and skilled nursing facility, and physician). | Beneficiaries with lower levels of continuity of care were younger, had a higher income, and more comorbid medical conditions. Lower continuity of care is associated with higher rates of hospitalization, emergency department visits, testing, and health care spending. Almost 50% of patients had at least 1 hospitalization and emergency department visit during the year. Utilization was lower with increasing level of continuity. |
| Aaron Jones et al., 2020(59) | Retrospective Cohort | Community-dwelling older adults with complex care needs | Measured continuity of primary and specialty physician care over two years prior to a home care assessment. Categorized into low, medium, and high groups based on terciles of the distribution. | None | Emergency department visits and hospital admissions within six months of assessment. | Higher continuity of both primary and specialty physician care was independently associated with reduced risks of emergency department visits (HR for primary care = 0.90 [0.89-0.92], HR for specialty care = 0.93 [0.91-0.95]) and hospital admissions (HR for primary care = 0.94 [0.92-0.96], HR for specialty care = 0.92 [0.90-0.94]). The effect of continuity of specialty care was stronger among patients who saw four or more physician specialties. |
| Almaawiy et al., 2014(53) | Retrospective population-based cohort study | Cancer patients at end-of-life | Family physician continuity of care assessed using Modified Usual Provider of Care score and visits/week | None (Observational Study) | Location of death, hospital and emergency department visits in the last 2 weeks of life. | Increased continuity of care from family physicians is associated with decreased odds of hospital death and acute care visits in the last 2 weeks of life. More visits per week generally associated with better outcomes, except when exceeding 4 visits/week. |
| Nyweide & Bynum, 2017(61) | Survival Analysis | Risk of ED Episodes | Higher continuity in ambulatory care (measured using COC and UPC scores) | None | Rate of ED episodes, type of ED episode | - A 1% decrease in the rate of ED episodes for every 0.1-point increase in COC score (adjusted HR 0.99; 95% CI 0.99 to 0.99; P<.001). <br>- A 2% decrease for every 0.1-point increase in UPC score (adjusted HR 0.98; 95% CI 0.98 to 0.99; P<.001). <br>- Higher continuity associated with a 1% lower risk of observation stay but a 3% to 4% higher risk of hospital admission relative to an ED visit with discharge home. |
| Gallo et al., 2013(60) | Long term follow-up of multi-site practice RCT | Major and minor depression in older adults | For two years, a depression care manager worked with primary care physicians in intervention practices to provide algorithm-based care for depression, including psychotherapy, medication adjustments, and monitoring. | Usual care | Mortality risk based on a median follow-up of 98 months | Patients with major depression in intervention practices had a 24% lower risk of mortality compared to usual care (HR 0.76, 95% CI 0.57 to 1.00; P=0.05). No significant effect on mortality was found for minor depression. |
| Menec et al., 2006(63) | Observational Survey | N/A (General older adult population) | High Continuity of Care with Family Physician | Low Continuity of Care with Family Physician | Hospitalizations (for ambulatory care-sensitive conditions and all conditions) | High continuity of care was associated with reduced odds of ambulatory care-sensitive hospitalizations (adjusted odds ratio = 0.67, 95% CI 0.51-0.90) but not related to hospitalizations for all conditions. The study underscores the importance of continuity of primary care in reducing potentially avoidable hospitalizations among older adults. |
| Serina et al., 2023(62) | Multiple methods | Sensory impairments, cognitive impairments, mobility challenges, receiving end-of-life care in older adults | Telehealth | None | Physician perceptions on the efficacy of telehealth for special populations of older adults | - 50% of emergency physicians, 33% of geriatricians, and 18% of primary care physicians viewed telehealth as a poor substitute for providing end-of-life care. - For hearing, vision, and cognitive impairments, 61%, 58%, and 54%, respectively, viewed telehealth as a good or fair substitute. - 98% indicated that telehealth was a good or fair substitute for in-person care for those with mobility impairment (p < .001). |
| Lan & Chen, 2022(64) | Quantitative, cross-sectional | Diabetes or Hypertension | Use of a telehealth care system by patients in remote areas | None | Satisfaction, attitudes, and continued use of telehealth systems. Information, system, and service quality as influenced by perceived ease of use and perceived usefulness of the technology acceptance model. | Information, system, and service quality were significantly influenced by the mediating effect of perceived ease of use and perceived usefulness. The use of telehealth care systems among chronic patients increased significantly. |
