# Supplementary figures and images for: Continuity and sustainability of care in family medicine: Assessing its association with quality of life and health outcomes in older populations—A systematic review
Source: PLoS One. 2024 Dec 23;19(12):e0299283. doi: 10.1371/journal.pone.0299283 (PMC11666006; doi:10.1371/journal.pone.0299283)

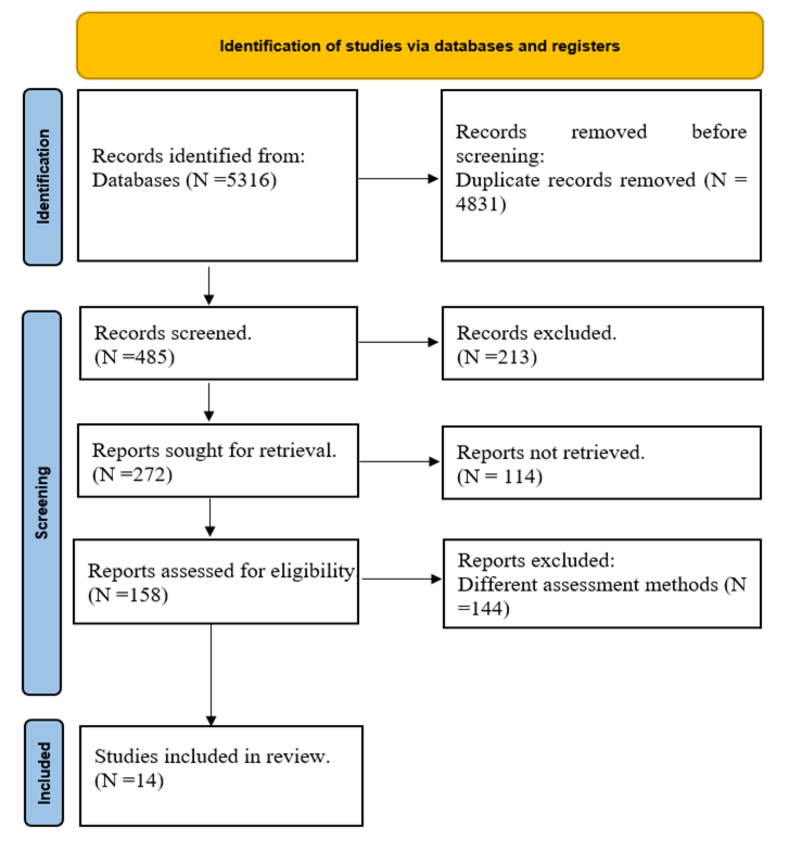

Supplement: S1 Fig — (TIF) [file pone.0299283.s002.tif]

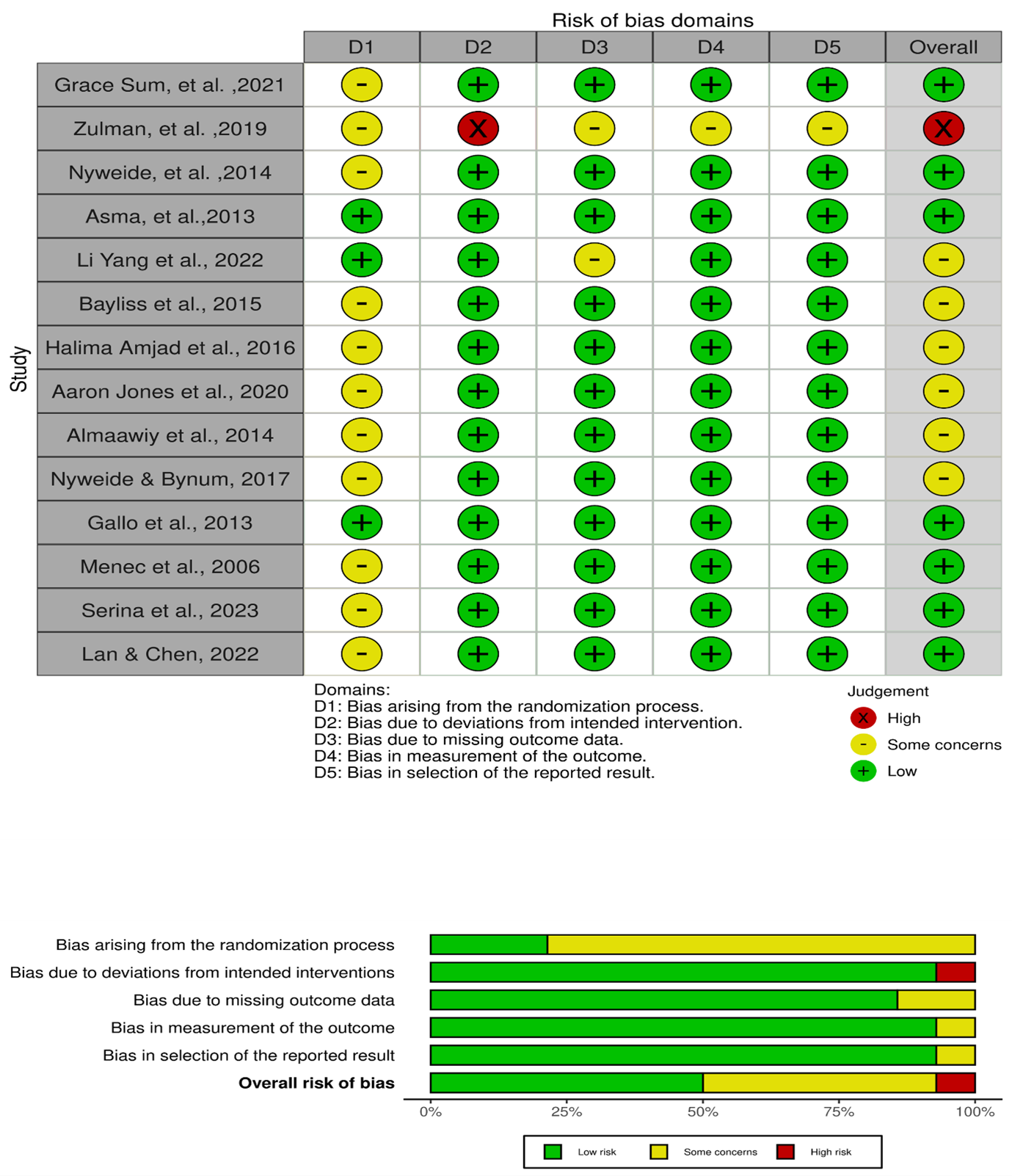

Supplement: S2 Fig — (TIF) [file pone.0299283.s003.tif]
