# Supplementary material for: Continuity and sustainability of care in family medicine: Assessing its association with quality of life and health outcomes in older populations—A systematic review
Source: PLoS One. 2024 Dec 23;19(12):e0299283. doi: 10.1371/journal.pone.0299283 (PMC11666006; doi:10.1371/journal.pone.0299283)
Supplement: Attachment — Submitted filename: Response to Reviewers.docx [file pone.0299283.s004.docx]

**Rebuttal Letter**

**To the Editorial Team and Reviewers of PLOS ONE,**

**Subject: Response to Reviewers' Comments - Manuscript ID: PONE-D-24-05114**

We deeply appreciate the time and effort the reviewers have invested in evaluating our manuscript titled "Continuity and Sustainability of Care in Family Medicine: Assessing Its Association with Quality of Life and Health Outcomes in Older Populations - A Systematic Review." We are grateful for the constructive feedback that has significantly contributed to enhancing the quality and clarity of our manuscript.

Below, we respond to each comment and detail the revisions made to the manuscript in accordance with the suggestions provided.

**Response to Reviewer #1 Comments:**

We thank Reviewer #1 for acknowledging the manuscript's well-articulated nature and its contribution to the existing body of knowledge. We also appreciate the constructive suggestion to provide more details on the sections with significant potential biases.

*Comment:* Desire for more information on sections with significant potential biases.

*Response:* In response to this insightful comment, we have carefully reviewed our manuscript and added a new subsection titled "Assessment of Potential Biases in Reviewed Articles" within the "Methods" section. Here, we discuss in depth the recognized sources of potential bias in the included studies, such as selection bias, information bias, and any biases arising from study design. This enhancement aims to provide readers with a transparent overview of the methodological rigor of the studies we analyzed and the potential impact of these biases on our conclusions.

**Revised Sections:** "Methods > Assessment of Potential Biases in Reviewed Articles"

**Response to Reviewer #2 Comments:**

We are grateful to Reviewer #2 for recognizing the manuscript's contribution to understanding the correlation between continuity of care, quality of life, and health outcomes in older populations. We have addressed each point as follows:

*Comment:* Request for better alignment between the study's aim in the Abstract and the manuscript.

*Response:* We acknowledge this discrepancy and have carefully revised both the Abstract and the corresponding sections within the manuscript to ensure that the study's aim is consistently articulated. We believe that these revisions have improved the clarity and coherence of our manuscript.

**Revised Sections:** "Abstract" and "Introduction"

*Comment:* Suggestion to analyze continuity of care, quality of life, and health outcomes in the context of patient safety.

*Response:* This valuable suggestion prompted us to expand our discussion on the interconnectedness of continuity of care, quality of life, and patient safety. We have included a new subsection titled "Continuity of Care and Patient Safety" within the "Discussion" section. This addition explores how enhanced continuity of care can contribute to improved patient safety outcomes, including reduced medical errors and improved management of chronic conditions, thereby positively influencing quality of life and health outcomes in older populations.

**Revised Sections:** "Discussion > Continuity of Care and Patient Safety"

We hope that the revisions and responses adequately address the reviewers' concerns and improve the manuscript. We are enthusiastic about the opportunity to enhance our work based on such insightful feedback and believe the revised manuscript is now better positioned for publication in PLOS ONE.

Thank you for considering our manuscript for publication. We look forward to your feedback.

Kind regards,

Mohammed Nasser Albarqi, Ph.D.
